# The characteristics of adverse reactions of three anti-prostate cancer drugs based on Vigiaccess database and bibliometric analysis

**DOI:** 10.3389/fphar.2025.1570661

**Published:** 2025-03-27

**Authors:** Jianqing Wang, Jia You, Weixing Huang, Chiting Yuan, Jiangjie Chen, Feifei Wang, Wei Wang, Liwei Zhang

**Affiliations:** ^1^ Nephrology department, Taizhou Hospital of Zhejiang Province, Zhejiang University School of Medicine, Taizhou, Zhejiang, China; ^2^ School of Integrative Medicine, Nanjing University of Chinese Medicine, Nanjing, China; ^3^ Department of Nursing, The First Affiliated Hospital, Zhejiang University School of Medicine, Hangzhou, Zhejiang, China; ^4^ Department of General surgery, Taizhou Hospital of Zhejiang Province, Zhejiang University School of Medicine, Taizhou, China; ^5^ Department of Orthopedics, Taizhou Hospital of Zhejiang Province, Zhejiang University School of Medicine, Taizhou, China; ^6^ Institute of Bone Metabolism, Taizhou Hospital of Zhejiang Province, Zhejiang University School of Medicine, Taizhou, China

**Keywords:** prostate cancer, adverse drug reactions, WHO-vigiaccess, androgen antagonists, apalutamide, darolutamide, enzalutamide

## Abstract

**Background:**

Androgen antagonists, including apalutamide, darolutamide, and enzalutamide, play a crucial role in the treatment of prostate cancer. This research evaluated the adverse drug reactions (ADRs) associated with the use of these androgen antagonists as reported by the World Health Organization (WHO). Additionally, it compared the adverse drug reaction (ADR) profiles of the three drugs to identify which one presents the lowest risk for individualized patient use.

**Methods:**

This study employed a retrospective descriptive analysis design. We collected adverse event reports for three marketed androgen antagonists from WHO-VigiAccess and analyzed them in combination with a bibliometric analysis. We calculated the percentage of adverse reactions reported for each drug to compare the similarities and differences in adverse reactions among the three drugs.

**Results:**

A total of 172,020 adverse events (AEs) associated with three androgen antagonists were reported in VigiAccess at the time of this study. Our findings show apalutamide causes the most endocrine disorders. Darolutamide has the highest rate of blood and lymphatic disorders, while enzalutamide causes the most nervous system disorders. The ten most common ADRs identified were fatigue, rash, death, hot flush, diarrhoea, asthenia, nausea, dizziness, arthralgia, and decreased appetite.

**Conclusion:**

This study utilizes real data from WHO-VigiAccess, which offers valuable insights for clinical reference. On one hand, we confirm both existing and potential adverse effects associated with androgen antagonists. On the other hand, We analyzed the possible future research directions, thereby supporting the case for more scientific treatment.

## Introduction

Androgens play a vital role in preserving typical male physiological functions and sexual differentiation, which is contained in the prostate gland (Werner and Holterhus, 2014). Furthermore, emerging evidence highlights their ​critical involvement in non-metastatic castration resistance to diseases such as prostate cancer. Androgens activate a series of signaling pathways by binding to androgen receptors, thereby affecting tumor growth (Mills, 2014). The activation of androgen signaling pathway promotes the growth of prostate cancer cells, while promoting tumor invasion and metastasis (Kim et al., 2022). Drugs that target androgen receptors have been used to inhibit the cancer-promoting effects of androgen signaling pathways. The SPARTAN, ARAMIS, and PROSPER studies demonstrated that androgen receptor inhibitors (apalutamide, darolutamide, enzalutamide) can extend metastasis-free survival and overall survival in nonmetastatic castration-resistant prostate cancer (nmCRPC) patients with a brief prostate-specific antigen (PSA) doubling time. These antiandrogen medications have been incorporated into clinical practice as a novel standard of care (Cattrini et al., 2022). For many years, patients with advanced prostate cancer were primarily treated with androgen deprivation therapy (ADT). However, patients with metastatic hormone-sensitive prostate cancer (mHSPC) who receive ADT only have a high risk of developing metastatic castration-resistant prostate cancer (mCRPC) (Xia et al., 2023). While androgen antagonists are generally well tolerated and clinically proven to be effective. For example, In a double-blind, phase 3 trial, androgen-deprivation therapy combined with apalutamide has been confirmed to prolong the survival of patients with metastatic and castration-sensitive prostate cancer without significant increase in adverse events (AEs) (Chi et al., 2019).

We searched the Drugs website (https://www.drugs.com/) to learn more about apalutamide, darolutamide, enzalutamide and their reported adverse reactions. The three drugs are oral androgen receptor inhibitors used to treat prostate cancer at different stages. Apalutamide is approved for nmCRPC and metastatic castration-sensitive disease (mCSPC) when combined with ADT. Darolutamide is indicated for nmCRPC and mHSPC alongside ADT or docetaxel, leveraging its unique structure to minimize central nervous system (CNS) penetration. Enzalutamide, a first-generation agent, is used for nmCRPC, mCRPC, and mHSPC with ADT. Enzalutamide is frequently associated with fatigue, hypertension, dizziness, and CNS-related issues, likely due to its ability to cross the blood-brain barrier. Apalutamide shares similar CNS risks but may also cause rash, hypothyroidism, and joint pain. Darolutamide, with limited CNS penetration, exhibits fewer neurological side effects but has been linked to fatigue, nausea, and elevated liver enzymes. All three agents may increase cardiovascular risks, though enzalutamide and apalutamide carry stronger warnings. Gastrointestinal disturbances are common across all, while enzalutamide and apalutamide are more often tied to seizures and thyroid dysfunction. Darolutamide’s distinct profile may reduce drug interactions compared to the others. Despite stringent pre-marketing requirements, the actual safety of drugs still needs to be verified by analysis of a large amount of data, especially for biologics. Therefore, it is necessary to further study the adverse reactions of androgen antagonists.

WHO-VigiAccess is a free portal to the PIDM database, enabling access to safety reports of medicinal products received by the UMC. It was launched by the World Health Organization (WHO) in 2015 to provide public access to information in VigiBase, the WHO global database of reported potential side effects of medicinal products. These side effects, known technically as adverse drug reactions (ADRs) and adverse events following immunization (AEFIs), are reported by national pharmacovigilance centres or national drug regulatory authorities that are members of the WHO Programme for International Drug Monitoring (PIDM) (Watson et al., 2018; Habarugira and Figueras, 2021).

This study looked for three androgen antagonists approved by the WHO: apalutamide, darolutamide, enzalutamide with similar efficacy. The three drugs for the treatment of nmCRPC have been studied over the past decade. Clinicians often need to develop personalized treatment plans based on adverse drug reactions. This study conducted a comparative pharmacovigilance analysis to evaluate disproportionality in adverse drug reaction (ADR) profiles among three therapeutic agents, utilizing safety surveillance data from the WHO-VigiAccess global database.

## Materials and methods

### Data source

We use WHO-VigiAccess to retrieve all reported adverse events following the administration of androgen antagonists. The login URL is https://www.vigiaccess.org. Data were collected based on age groups, sex, report year, and continents through WHO-VigiAccess. Descriptive statistics were generated using Excel 2019. The classification of adverse events in WHO-VigiAccess is based on system organ class (SOC) and preferred terms (PTs) from the Medical Dictionary for Regulatory Activities (MedDRA). The MedDRA terms were derived from various sources, including the WHO Adverse Reaction Terminology (WHO-ART). Records for androgen antagonists were retrieved, and individual AEs were identified at the MedDRA SOC and PT levels to describe the spectrum of toxicities. A total of 27 items were categorized by SOC, and 20 items related to disease symptoms were selected for analysis (Brown et al., 1999). This study focused on the PTs, which are the level used in the publicly accessible VigiBase database via WHO-VigiAccess. We pay particular attention to serious adverse events, including treatment-interrupted events such as death, hospitalization and disability. It is important to note that due to strict data protection laws and agreements, individual case safety reports cannot be viewed in Vigiaccess. In order to carry out bibliometric analysis,we exported androgen antagonist-related literature from the Web of Science (WoS) database, constructed a co-occurrence network, performed cluster analysis, and ultimately conducted visualization processing.

### Data extraction and mining

We use common names (apalutamide, darolutamide, enzalutamide) to identify related reports. In general, the drugs reported in WHO-VigiAccess fall into four categories: primary suspect drugs, secondary suspect drugs, companion drugs, and interacting drugs. In the search results, we screened the reports with the target drug as the primary suspect drug and excluded duplicate reports and invalid reports. We performed a statistical analysis of the first suspect drug reports after screening. We first analyzed the clinical characteristics of these trials, including demographic characteristics (age, sex) and reporting characteristics (reporting year, region). Based on the MedDRA terminology system, we analyzed the distribution and characteristics of PTs within their respective SOC hierarchies. Severe consequences, meanwhile, were defined as death and hospitalization. After exporting the literature, we performed data deduplication, standardization, and completion to ensure data quality.

### Statistical analysis

In this study, a retrospective descriptive approach was employed. The analysis involved comparing drugs based on the quantity and rate of ADR reports. At the same time, we used statistical methods such as reporting Odds ratio method (ROR) and Proportional Reporting ratio method (PRR) to mining the adverse event signal of the first suspected drug. The following approaches can be utilized to assess the degree of statistical correlation between a medication and an adverse reaction, which in turn can further substantiate the drug’s status as the primary suspect. The Reporting Ratio technique gauges the intensity of the link between a drug and an adverse event by contrasting the incidence of a specific drug-adverse event pair with that of other drug-adverse event pairs (Rothman et al., 2004). The Proportional Reporting Ratio (PRR) method evaluates the link between a drug and an adverse event. It does this by computing the ratio of the target drug to the target adverse event, as well as the ratio of the non-target drug to the target adverse event (Evans et al., 2001). The Bayesian Confidence Propagation Neural Network (BCPNN) is a signal detection approach grounded in Bayesian statistical principles. It is utilized to analyze data within adverse drug reaction monitoring databases, aiming to pinpoint potential adverse drug reaction signals (Bate et al., 1998). In the BCPNN, Information Component (IC) is the core indicator used to measure the degree of association between a drug and an adverse reaction (Bate, 2007). Empirical Bayesian Geometric Mean (EBGM) is another signal detection method based on Bayesian methods, which assesses the association between drugs and adverse events by calculating the ratio between the number of reported drugs and adverse events and the number of expected reports (Noguchi et al., 2021). These methods have important application value in the detection of adverse drug reaction signals, and can help identify the potential association between drugs and adverse events, so as to improve the monitoring and evaluation of drug safety (Table 1).

**TABLE 1:** Four major algorithms used for signal detection.

Algorithms	Calculation formulas	Criteria
ROR	ROR= $\frac{\left(a/c\right)}{\left(b/d\right)}$ = $\frac{ad}{bc}$	95%CI>1
	95%CI= $e^{\ln\left(ROR\right)\pm 1.96\sqrt{\left(\frac{1}{a}+\frac{1}{b}+\frac{1}{c}+\frac{1}{d}\right)}}$	
PRR	PRR= $\frac{a/\left(a+b\right)}{c/\left(c+d\right)}$	PRR≥2, 95%CI>1
	95%CI= $e^{\ln\left(PRR\right)\pm 1.96\sqrt{\left(\frac{1}{a}+\frac{1}{b}+\frac{1}{c}+\frac{1}{d}\right)}}$	
BCPNN	IC= $\log_{2}\frac{a\left(a+b+c+d\right)}{\left(a+b\right)\left(a+c\right)}$	IC025>0
	E(IC)= $\log_{2}\frac{\left(a+\gamma 11\right)\left(a+b+c+d+\alpha \right)\left(a+b+c+d+\beta \right)}{\left(a+b+c+d+\gamma \right)\left(a+b+\alpha 1\right)\left(a+c+\beta 1\right)}$	
EBGM	EBGM= $\frac{a\left(a+b+c+d\right)}{\left(a+c\right)\left(a+b\right)}$	EBGM05>2
	95%CI= $e^{\ln\left(EBGM\right)\pm 1.96\sqrt{\left(\frac{1}{a}+\frac{1}{b}+\frac{1}{c}+\frac{1}{d}\right)}}$	

## Results

### Description of the studied cases

**FIGURE 1:**
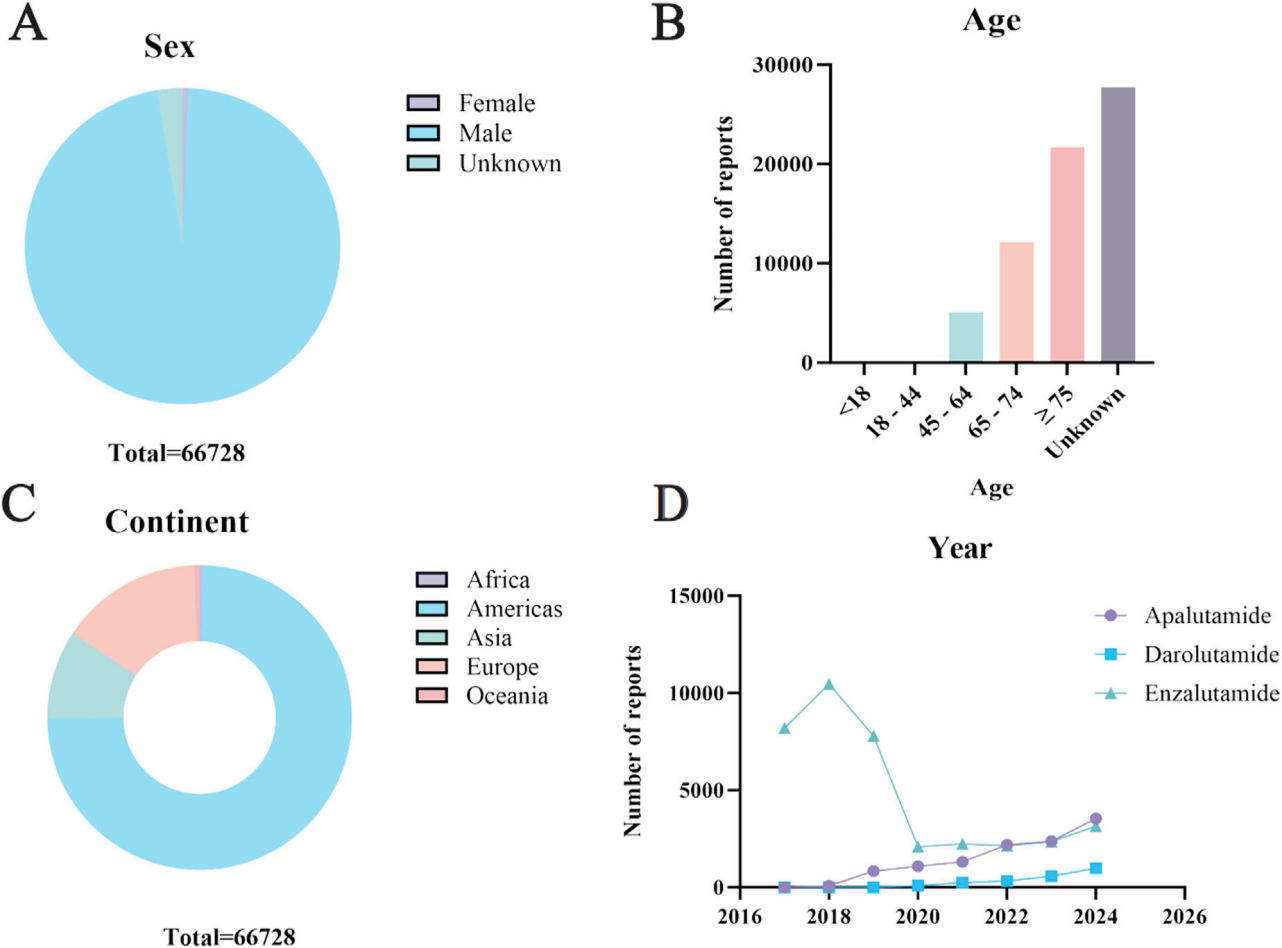
Characteristics of ADR reports of three androgen antagonists **(A)** Gender distribution of ADR report **(B)** Age distribution of ADR reports **(C)** Continent distribution of ADR reports **(D)** Trend of annual report volume.

Apalutamide and darolutamide were collected by WHO-VigiAccess from 2016 to 2024 for 11,452 and 2,269 adverse reaction reports, respectively. Enzalutamide had the highest number of adverse reactions reported, with 53,007 cases reported from 2012 to 2024. A total of 66,728 cases were reported for these three drugs (Table 2). In the 172,020 ADR reports concerning the three androgen antagonist drugs, there were 20,965 cases related to apalutamide, 5,236 cases to darolutamide, and 145,819 cases to enzalutamide. Except for 355 cases reported as female, all were male (Table 3). Excluding reports of unknown age, incidence increased with age group. Among the top five reporting countries, the Americas region has the highest incidence of adverse reactions, while Africa has the lowest (Figure 1).

**TABLE 2:** Number and distribution of ADR reports of three androgen antagonists.

	Apalutamide	Darolutamide	Enzalutamide
Number of ADR reports	11,452	2,269	53,007
Sex			
Female	35 (0.31%)	7 (0.31%)	313 (0.59%)
Male	10,751 (93.88%)	2,124 (93.61%)	51,764 (97.66%)
Unknown	666 (5.82%)	138 (6.08%)	930 (1.75%)
Age			
<18	3 (0.03%)	2 (0.09%)	7 (0.01%)
18–44	10 (0.09%)	3 (0.13%)	58 (0.11%)
45–64	722 (6.30%)	262 (11.55%)	4,091 (7.72%)
65–74	2,217 (19.36%)	495 (21.82%)	9,422 (17.78%)
≥75	3,624 (31.65%)	777 (34.24%)	17,308 (32.65%)
Unknown	4,876 (42.58%)	730 (32.17%)	22,121 (41.73%)
Top 5 reporting countries			
Africa	141 (1.23%)	0 (0.00%)	65 (0.12%)
Americas	5,932 (51.80%)	1,127 (49.67%)	42,759 (80.67%)
Asia	1,639 (14.31%)	368 (16.22%)	4,220 (7.96%)
Europe	3,724 (32.52%)	718 (31.64%)	5,648 (10.66%)
Oceania	16 (0.14%)	56 (2.47%)	315 (0.59%)
Reporting year			
Before 2017	3 (0.03%)	2 (0.09%)	14,551 (27.45%)
2017	4 (0.03%)	2 (0.09%)	8,209 (15.49%)
2018	75 (0.65%)	0 (0.00%)	10,471 (19.75%)
2019	844 (7.37%)	11 (0.48%)	7,789 (14.69%)
2020	1,091 (9.53%)	97 (4.28%)	2094 (3.95%)
2021	1,317 (11.50%)	242 (10.67%)	2,238 (4.22%)
2022	2,191 (19.13%)	336 (14.81%)	2,140 (4.04%)
2023	2,385 (20.83%)	580 (25.56%)	2,361 (4.45%)
2024	3,542 (30.93%)	999 (44.03%)	3,154 (5.95%)

**TABLE 3:** ADR number and Incidence rate of SOCs of three androgen antagonists.

System organ classes	Apalutamide (N = 20,965)	Darolutamide (N = 5,236)	Enzalutamide (N = 145,819)
Blood and lymphatic system disorders	280 (1.34%)	113 (2.16%)	1,205 (0.83%)
Cardiac disorders	486 (2.32%)	119 (2.27%)	2,241 (1.54%)
Congenital, familial and genetic disorders	7 (0.03%)	1 (0.02%)	30 (0.02%)
Ear and labyrinth disorders	90 (0.43%)	22 (0.42%)	715 (0.49%)
Endocrine disorders	195 (0.93%)	5 (0.10%)	98 (0.07%)
Eye disorders	133 (0.63%)	55 (1.05%)	1,661 (1.14%)
Gastrointestinal disorders	1,328 (6.33%)	469 (8.96%)	14,604 (10.02%)
General disorders and administration site conditions	3,835 (18.29%)	992 (18.95%)	35,797 (24.55%)
Hepatobiliary disorders	103 (0.49%)	85 (1.62%)	461 (0.32%)
Immune system disorders	83 (0.40%)	19 (0.36%)	328 (0.23%)
Infections and infestations	586 (2.80%)	142 (2.71%)	4,007 (2.75%)
Injury, poisoning and procedural complications	1,443 (6.88%)	366 (6.99%)	10,731 (7.36%)
Investigations	1,482 (7.07%)	512 (9.78%)	10,219 (7.01%)
Metabolism and nutrition disorders	447 (2.13%)	111 (2.12%)	4,709 (3.23%)
Musculoskeletal and connective tissue disorders	973 (4.64%)	370 (7.07%)	10,467 (7.18%)
Neoplasms benign, malignant and unspecified (incl cysts and polyps)	604 (2.88%)	246 (4.70%)	7,216 (4.95%)
Nervous system disorders	1,495 (7.13%)	433 (8.27%)	15,569 (10.68%)
Psychiatric disorders	514 (2.45%)	147 (2.81%)	5,887 (4.04%)
Renal and urinary disorders	326 (1.56%)	127 (2.43%)	2,645 (1.81%)
Reproductive system and breast disorders	132 (0.63%)	57 (1.09%)	785 (0.54%)
Respiratory, thoracic and mediastinal disorders	574 (2.74%)	172 (3.29%)	4,407 (3.02%)
Skin and subcutaneous tissue disorders	4,078 (19.45%)	369 (7.05%)	4,196 (2.88%)
Social circumstances	40 (0.19%)	17 (0.33%)	356 (0.24%)
Surgical and medical procedures	594 (2.83%)	83 (1.59%)	1,208 (0.83%)
Vascular disorders	1,056 (5.04%)	199 (3.80%)	4,884 (3.35%)
Product issues	81 (0.39%)	5 (0.10%)	1,391 (0.95%)

### Adverse drug reaction distribution and signal distribution of three androgen antagonists at SOC level

In this study, adverse reactions to three androgen antagonists included 27 SOCs, based on analysis of adverse event reports. Due to incomplete statistics of Pregnancy, puerperium and perinatal conditions, only 26 cases of SOC were actually analyzed. All three drugs had a high rate of general disorders and administration site conditions, as well as gastrointestinal disorders. These three drugs also showed a high incidence rate of skin and subcutaneous tissue disorders, with apalutamide (19.45%) having the highest rate. Enzalutamide had the highest rate of nervous system disorders (10.68%), while darolutamide had the highest rate of blood and lymphatic system disorders (2.16%). We found that apalutamide is the most likely to develop endocrine disorders (0.93%) (Table 3). The results of ADR signal detection were consistent with the incidence rate. Previous studies have shown that common endocrine adverse events associated with apalutamide include hypothyroidism, high blood pressure, and rashes (Pyrgidis et al., 2021). Darolutamide is more associated with hepatobiliary disorders, and enzalutamide has the strongest signal of product issues (Supplementary Table S1).

### Distribution of adverse drug reactions of three androgen antagonists at PT level

We calculated the proportion of each adverse reaction by the number of ADR reports for the three drugs. At PT level, the common ADRs in the three drugs are fatigue, rash, death, hot flush, diarrhoea, asthenia, nausea, dizziness, arthralgia and decreased appetite. The proportion of apalutamide-related rash was significantly higher than that of the other two androgen antagonists, while the proportion of fatigue related to darolutamide and enzalutamide was significantly higher than that of apalutamide (Table 4).

**TABLE 4:** Top 20 ADRs of three androgen antagonists.

Apalutamide		Darolutamide		Enzalutamide	
ADR	Report rate %	ADR	Report rate %	ADR	Report rate %
Rash	9.93	Fatigue	6.26	Fatigue	6.34
Fatigue	4.25	Rash	2.46	Death	4.75
Death	4.17	Off label use	2.39	Malignant neoplasm progression	3.37
Hot flush	2.59	Death	2.23	Drug ineffective	2.63
Product dose omission issue	1.77	Asthenia	2.18	Prostatic specific antigen increased	2.53
Diarrhoea	1.72	Diarrhoea	1.83	Asthenia	2.48
Pruritus	1.47	Hot flush	1.83	Decreased appetite	2.09
Hypertension	1.46	Prostatic specific antigen increased	1.53	Nausea	2.06
Arthralgia	1.45	Pain in extremity	1.45	Hot flush	1.98
Asthenia	1.42	Nausea	1.43	Dizziness	1.86
Prostatic specific antigen increased	1.33	Dizziness	1.28	Diarrhoea	1.72
Dizziness	1.23	Arthralgia	1.20	Back pain	1.35
Off label use	1.18	Dyspnoea	1.15	Underdose	1.34
Fall	1.17	Decreased appetite	1.07	Arthralgia	1.22
Drug ineffective	1.06	Neuropathy peripheral	1.03	Constipation	1.05
Decreased appetite	1.01	Product dose omission issue	0.94	Headache	1.04
Nausea	0.94	Hormone-refractory prostate cancer	0.94	Weight decreased	1.03
Rash pruritic	0.88	Hypertension	0.90	Fall	1.02
Weight decreased	0.83	Prostate cancer	0.84	Pain	0.94
Interstitial lung disease	0.77	Peripheral swelling	0.78	Malaise	0.87

### Serious AEs of three androgen antagonists drugs

**FIGURE 2:**
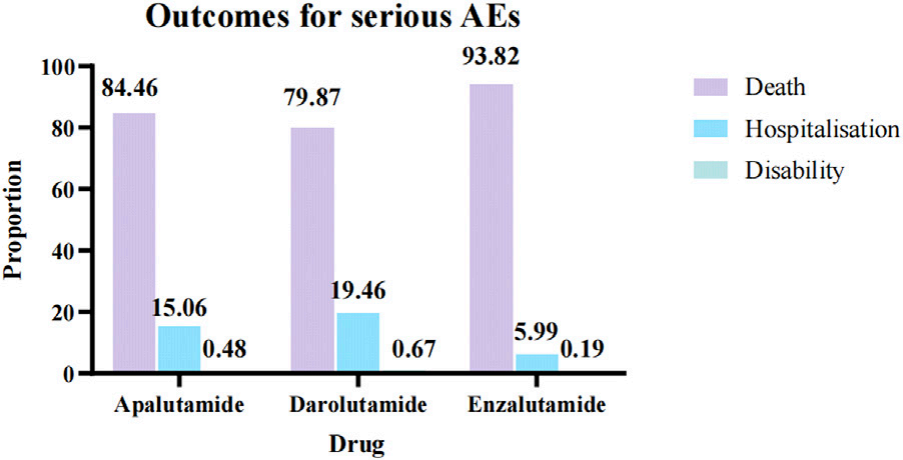
Serious AEs of three androgen antagonists drugs.

Through WHO-VigiAccess, we were also able to identify major adverse events involving androgen antagonists, including death, hospitalization, and disability, with death accounting for the highest proportion. The reported number and rate of major adverse reactions were apalutamide(20,965, 4.94%), darolutamide (5236,2.85%) and enzalutamide (145,819,5.06%), respectively. All three androgen antagonists had a low likelihood of causing disability. Enzalutamide is the most likely to cause serious adverse reactions and has the greatest probability of leading to death (Figure 2).

### The same and different points of common ADRs of three androgen antagonists

We compared and analyzed the similarities and differences of adverse reactions of three androgen antagonists. We found a total of 162 identical adverse reaction signals at the PT level and categorized them by system organ classes. The top five system organ classes with the most adverse reaction signals are general disorders and administration site conditions, nervous system disorders, gastrointestinal disorders, musculoskeletal and connective tissue disorders, skin and subcutaneous tissue disorders (Table 5).

**TABLE 5:** Same ADRs among three androgen antagonists.

System organ classes	ADRs	Signal N
General disorders and administration site conditions	Peripheral swelling, Gait inability, Pyrexia, Disease progression, Oedema, Chest discomfort, Swelling face, Chest pain, Asthenia, Adverse drug reaction, Drug ineffective, Drug intolerance, Gait disturbance, Death, General physical health deterioration, Oedema peripheral, Pain, Drug interaction, Condition aggravated, Chills, Malaise, Fatigue, Illness, Feeling hot, Feeling abnormal, Adverse event	25
Nervous system disorders	Taste disorder, Transient ischaemic attack, Dysgeusia, Somnolence, Loss of consciousness, Hypersomnia, Paraesthesia, Memory impairment, Ageusia, Dementia, Hypoaesthesia, Headache, Syncope, Neuropathy peripheral, Tremor, Cognitive disorder, Balance disorder, Cerebrovascular accident, Seizure, Dizziness, Lethargy	21
Gastrointestinal disorders	Abdominal distension, Vomiting, Diarrhoea, Abdominal discomfort, Dyspepsia, Abdominal pain upper, Dry mouth, Gastrooesophageal reflux disease, Gastrointestinal disorder, Nausea, Abdominal pain, Flatulence, Dysphagia, Constipation	14
Musculoskeletal and connective tissue disorders	Joint swelling, Arthralgia, Bone pain, Limb discomfort, Back pain, Mobility decreased, Muscle atrophy, Pain in extremity, Myalgia, Musculoskeletal pain, Muscle spasms, Muscular weakness, Arthritis	13
Skin and subcutaneous tissue disorders	Erythema, Urticaria, Alopecia, Dry skin, Night sweats, Rash pruritic, Pruritus, Skin exfoliation, Rash, Hyperhidrosis	10
Injury, poisoning and procedural complications	Product use in unapproved indication, Fracture, Off label use, Hip fracture, Incorrect dose administered, Fall, Wrong technique in product usage process, Inappropriate schedule of product administration, Product dose omission issue	9
Investigations	Weight decreased, Prostatic specific antigen increased, Blood glucose increased, Blood pressure decreased, Blood pressure increased, Heart rate increased, Weight increased, White blood cell count decreased, Haemoglobin decreased	9
Infections and infestations	Nasopharyngitis, Urinary tract infection, Pneumonia, Herpes zoster, Influenza, Infection, Sepsis, COVID-19	8
Renal and urinary disorders	Renal impairment, Acute kidney injury, Dysuria, Haematuria, Pollakiuria, Urinary retention, Nocturia, Renal failure	8
Psychiatric disorders	Insomnia, Hallucination, Anxiety, Depression, Eating disorder, Confusional state, Sleep disorder	7
Respiratory, thoracic and mediastinal disorders	Oropharyngeal pain, Dyspnoea, Pulmonary embolism, Interstitial lung disease, Epistaxis, Dyspnoea exertional, Cough	7
Cardiac disorders	Cardiac failure, Cardiac failure congestive, Cardiac disorder, Cardiac arrest, Myocardial infarction, Atrial fibrillation	6
Neoplasms benign, malignant and unspecified (incl cysts and polyps)	Metastases to bone, Hormone-refractory prostate cancer, Prostate cancer, Prostate cancer metastatic, Malignant neoplasm progression, Neoplasm malignant	6
Vascular disorders	Hot flush, Blood pressure fluctuation, Hypertension, Thrombosis, Hypotension	5
Blood and lymphatic system disorders	Neutropenia, Anaemia, Thrombocytopenia	3
Ear and labyrinth disorders	Tinnitus, Vertigo	2
Eye disorders	Visual impairment, Vision blurred	2
Metabolism and nutrition disorders	Dehydration, Decreased appetite	2
Reproductive system and breast disorders	Gynaecomastia, Erectile dysfunction	2
Hepatobiliary disorders	Hepatic function abnormal	1
Immune system disorders	Hypersensitivity	1
Surgical and medical procedures	Hospitalisation	1

**FIGURE 3:**
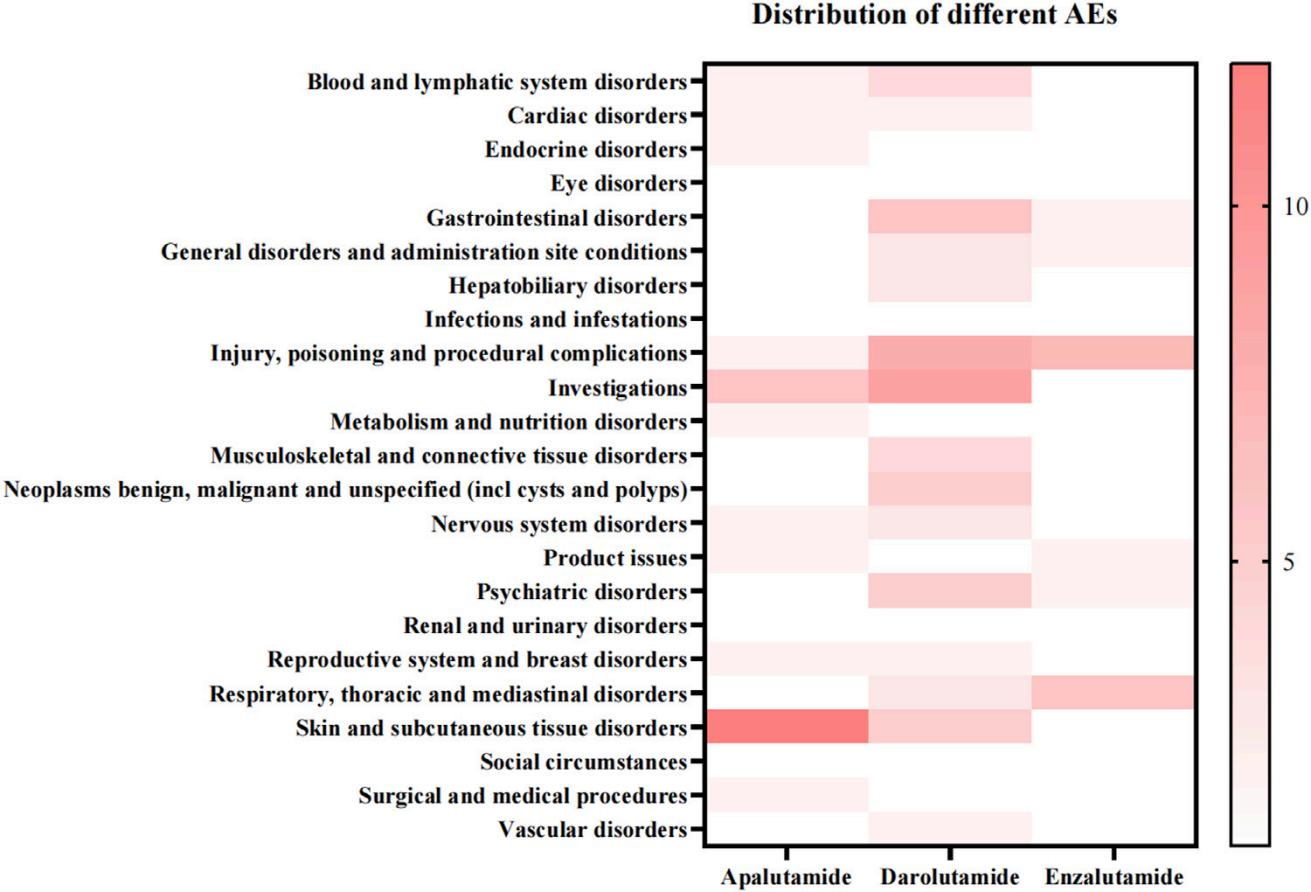
Distribution of different ADRs among three androgen antagonists.

According to the statistical results of different adverse reactions of the three drugs, darolutamide had the most adverse reactions, while enzalutamide had the least (Figure 3). We found that apalutamide has adverse effects such as hypothyroidism and thyroid disorder, while darolutamide may cause hepatic cytolysis, hypertransaminasaemia and adverse reactions such as hepatitis. It further verified the conclusion that apalutamide is more likely to cause endocrine disorders and darolutamide is more likely to cause hepatobiliary disorders. At the same time, apalutamide had the most specific adverse reactions in skin and subcutaneous tissue disorders, which was consistent with the conclusion that apalutamide had the highest incidence of rash. It is worth mentioning that the specific adverse reactions of darolutamide in vascular disorders include deep vein thrombosis and orthostatic hypotension, suggesting its cardiovascular adverse reactions (Table 6).

**TABLE 6:** Different ADRs among three androgen antagonists.

ADR	Apalutamide	Darolutamide	Enzalutamide
Blood and lymphatic system disorders	Eosinophilia, Agranulocytosis	Myelosuppression, Disseminated intravascular coagulation, Cytopenia, Blood disorder	
Cardiac disorders	Myocardial ischaemia, Acute myocardial infarction	Cardiotoxicity, Bradycardia	
Endocrine disorders	Hypothyroidism, Thyroid disorder		
Eye disorders		Blindness	Cataract
Gastrointestinal disorders	Rectal haemorrhage	Faeces soft, Frequent bowel movements, Colitis, Faeces discoloured, Gastrointestinal motility disorder, Swollen tongue	Retching, Gastrointestinal haemorrhage
General disorders and administration site conditions	Therapeutic product effect decreased	Pre-existing condition improved, Feeling cold, Exercise tolerance decreased	Treatment failure, Influenza like illness
Hepatobiliary disorders		Hepatic cytolysis, Hypertransaminasaemia, Hepatitis	
Infections and infestations	Lower respiratory tract infection	*Escherichia* infection	
Injury, poisoning and procedural complications	Incorrect dosage administered, Intentional product use issue	Limb injury, Toxicity to various agents, Incorrect product administration duration, Multiple fractures, Product prescribing issue, Product dose omission in error, Accidental exposure to product, Nerve injury	Product storage error, Intentional product misuse, Overdose, Underdose, Circumstance or information capable of leading to medication error, Intentional dose omission, Foreign body in throat
Investigations	Glycosylated haemoglobin increased, Blood potassium increased, Blood triglycerides increased, Blood thyroid stimulating hormone increased, Laboratory test abnormal, Prostatic specific antigen decreased	Blood sodium decreased, Blood calcium decreased, Red blood cell count decreased, Transaminases increased, Blood bilirubin increased, Heart rate decreased, International normalised ratio increased, Blood potassium decreased, Blood testosterone decreased	Full blood count decreased
Metabolism and nutrition disorders	Hyperkalaemia, Hypercholesterolaemia	Increased appetite	Hypophagia
Musculoskeletal and connective tissue disorders	Osteoporosis	Rhabdomyolysis, Musculoskeletal discomfort, Musculoskeletal disorder, Groin pain	
Neoplasms benign, malignant and unspecified (incl cysts and polyps)	Lung neoplasm malignant	Bladder cancer, Neoplasm progression, Metastases to spine, Metastases to pelvis, Malignant melanoma	
Nervous system disorders	Sensory disturbance, Cerebral infarction	Head discomfort, Parkinson’s disease, Motor dysfunction, Neuralgia, Presyncope	Dyskinesia
Product issues	Product label issue, Product packaging quantity issue		Product availability issue, Product physical issue
Psychiatric disorders		Poor quality sleep, Delirium, Apathy, Stress, Personality change	Disorientation, Restlessness
Renal and urinary disorders	Micturition urgency	Urinary tract disorder	
Reproductive system and breast disorders	Pelvic pain, Breast enlargement	Breast swelling, Breast pain	
Respiratory, thoracic and mediastinal disorders		Pulmonary hypertension, Lung disorder, Pneumonitis	Choking, Pleural effusion, Throat irritation, Chronic obstructive pulmonary disease, Rhinorrhoea, Productive cough
Skin and subcutaneous tissue disorders	Lichenoid keratosis, Skin toxicity, Drug reaction with eosinophilia and systemic symptoms, Erythema multiforme, Toxic epidermal necrolysis, Eczema, Dermatitis allergic, Stevens-johnson syndrome, Toxic skin eruption, Dermatitis, Skin reaction, Dermatitis exfoliative generalised	Haemorrhage subcutaneous, Skin atrophy, Palmar-plantar erythrodysaesthesia syndrome, Angioedema, Nail disorder	
Social circumstances		Bedridden	
Surgical and medical procedures	Therapy change, Surgery	Cardiac operation	
Vascular disorders		Deep vein thrombosis, Orthostatic hypotension	

### The research trend of androgen antagonists based on bibliometric analysis

**FIGURE 4:**
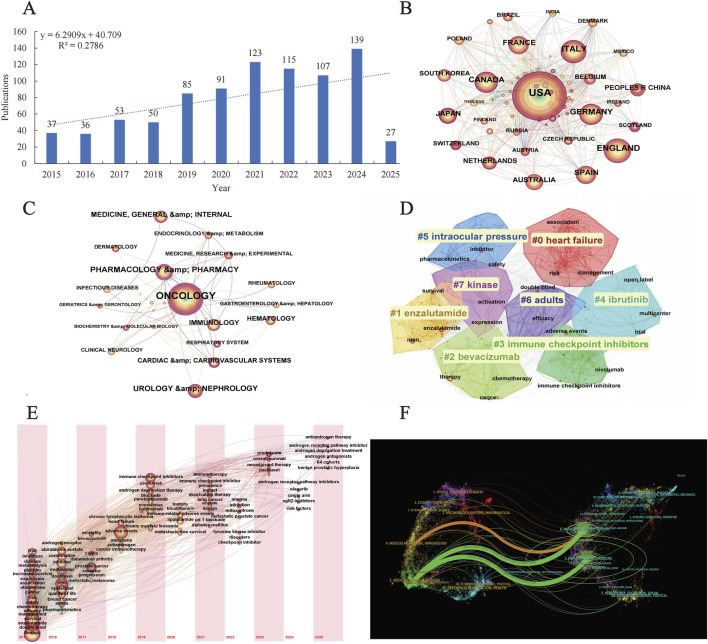
Bibliometric analysis **(A)** Annual publication trends **(B)** International collaboration networks **(C)** Keyword co-occurrence **(D)** Keyword clustering analysis **(E)** Keyword timezone map **(F)** Dual-map overlay of cross-disciplinary citation trajectories.

The bibliometric analysis revealed a consistent upward trend in the annual publication volume of androgen antagonists over the past decade, indicating intensified research focus and expanded therapeutic exploration in this field. Geographically, the majority of publications originated from the United States and select European nations, aligning with Vigiaccess database findings that identified the highest rate of adverse event reports from the Americas. Discipline co-occurrence mapping demonstrated strong associations between Oncology and Cardiovascular Systems as well as Geriatrics & Gerontology, highlighting the critical need to address cardiovascular toxicity associated with androgen antagonist therapy and the unique therapeutic requirements of elderly cancer patients. Journal distribution analysis further underscored the predominance of oncology- and pharmacy-focused periodicals, reinforcing the central role of androgen antagonists in prostate cancer management. Keyword cluster analysis revealed significant attention to heart failure risk and its clinical management, emphasizing the cardiovascular toxicity profile as a critical safety consideration for these agents. Temporal keyword evolution mapping delineated the strong association between androgen receptor pathway inhibitors and therapeutic strategies such as abiraterone and antiandrogen therapy, consolidating prostate cancer treatment as the primary research trajectory (Figure 4).

## Discussion

Apalutamide, darolutamide, and enzalutamide are next-generation androgen receptor signaling inhibitors (ARSIs) that target the androgen receptor (AR) pathway through distinct mechanisms, improving survival in nmCRPC. Apalutamide, a thiohydantoin derivative, inhibits AR nuclear translocation and DNA binding to androgen response elements (AREs), preventing transcriptional activation of androgen-dependent genes. Similarly, enzalutamide competitively antagonizes androgen binding to AR, blocks nuclear translocation, and disrupts AR-DNA interaction, thereby suppressing downstream signaling. Both agents exhibit no significant agonist activity but demonstrate central nervous system (CNS) penetration, contributing to seizure risk. In contrast, darolutamide, a structurally unique nonsteroidal AR antagonist with a polar pyrazole backbone, potently inhibits androgen binding while maintaining antagonistic efficacy even in AR-overexpressing cells. Its reduced blood-brain barrier penetration minimizes CNS-related adverse events. Pharmacokinetically, darolutamide undergoes CYP3A4-mediated metabolism with limited drug-drug interaction (DDI) potential, whereas apalutamide and enzalutamide act as strong CYP3A4 inducers, increasing DDI risks. While effective and generally tolerated, the mechanisms of action may raise safety concerns, highlighting the importance of studying their adverse effects and strengthening relevant management to improve therapeutic effect (Gasperoni et al., 2024).

Our study comprehensively evaluated the adverse events associated with three androgen antagonists (apalutamide, darolutamide, enzalutamide). By analyzing data from WHO-VigiAccess, the study confirmed previous findings of adverse reactions on drug labels, including common ADRs such as rashes. In addition, adverse reactions not indicated on the label, including elevated prostate-specific antigens, were also found. These findings highlight in particular the need for drug surveillance. At the beginning of treatment, potential adverse effects should be identified and effectively managed.

In terms of the total number of adverse reactions reported, the number of adverse reactions reported by enzalutamide was much higher than that of apalutamide and darolutamide. However, this does not necessarily mean that enzalutamide has a higher rate of adverse reactions or is less safe, and may be attributed to the much higher use of enzalutamide than the other two drugs. The significantly higher incidence of adverse reactions in men than in women may be attributed to the fact that androgen antagonists are primarily used to treat male prostate cancer (Al-Salama, 2018). The number of adverse reactions increased with the increase of age group, which may be related to the decline of physical function and underlying diseases in the elderly (Gao et al., 2024). The decrease in the number of adverse reactions reported with enzalutamide from 2017 to date and the increase in apalutamide and darolutamide indicate a change in clinical use (Desai et al., 2021). In fact, the AFFRIM trial found that men treated with enzalutamide reported higher rates of diarrhea, hot flashes, fatigue, high blood pressure, and a small percentage (0.6%) experienced seizures (Scher et al., 2012). Hypertension was a common adverse effect of enzalutamide in PREVAIL (Beer et al., 2014). A higher incidence of enzalutamide epilepsy was found in two subsequent phase 3 trials, ENZAMET and ARCHES (Davis et al., 2019; Armstrong et al., 2019). However, trials demonstrated that the adverse effects of apalutamide were milder, and adverse events such as dalloutamide seizures were less common (Rachner et al., 2018; Fizazi et al., 2019).

At the SOC level, the incidence of Skin and subcutaneous tissue disorders in apalutamide was much higher than that in darolutamide and enzalutamide. In one case report, four patients treated with apalutamide for castration-resistant prostate cancer developed severe and fatal drug eruptions including Stevens-Johnson syndrome and toxic epidermal necrolysis, with an average incubation period of 40 days. After discontinuation of apalutamide in all patients, three of them recovered (Wang et al., 2023). At the same time, apalutamide is more likely to cause heart and vessel disease. While enzalutamide had a higher incidence of nervous system disorders and psychiatric disorders than the other two drugs. Psychological adverse reactions may significantly affect patients. Such issues can impair both mental and physical wellbeing. They are often marked by feelings of low spirits, lack of energy, and sadness, which can further lead to sleep disturbances and a reduced ability to take pleasure in life (Cui, 2015). It is worth noting that clinical pharmacokinetic and pharmacodynamic analysis of darolutamide showed that darolutamide is over metabolized by oxidation and glucose-aldehyde acidification and excreted in urine and stool. Darolutamide should not be used in patients with moderate or severe renal or liver impairment (Podgoršek et al., 2023). The reason for the higher incidence of product issues with darolutamide may be attributed to its role as a substrate for P-gp and CYP3A4, as well as an inhibitor of breast cancer resistance protein (BCRP) and organic anion transporters (OATP1B1 and OATP1B3). This makes darolutamide prone to drug interactions when combined with other drugs, which can affect the metabolism and efficacy of the drug. Therefore, a thorough understanding of drug interactions is needed to optimize treatment outcomes and minimize adverse reactions (Bolek et al., 2024). At the PT level, the overall performance of darolutamide was relatively good in terms of adverse reactions, and the incidence of adverse reactions and serious adverse reactions was similar to enzalutamide, but the risk of treatment interruption due to adverse reactions was lower, and adverse reaction related mortality was slightly lower than enzalutamide. Apalutamide was slightly higher than the other two drugs in terms of risk of serious adverse reactions and treatment interruption.

The management of advanced prostate cancer with androgen antagonists (apalutamide, darolutamide, and enzalutamide) requires tailored strategies to mitigate their distinct adverse effect profiles. In view of the common situation of underlying diseases in the elderly, it is recommended to conduct a comprehensive assessment of liver and kidney function in elderly patients before medication. At the same time, consider the individual differences of the patient, such as body weight, to adjust the drug dose. When the elderly have basic diseases, try to reduce the variety of drugs and avoid unnecessary combination of drugs. The elderly are highly sensitive to the side effects of drugs, so the adverse reactions should be closely monitored and the medication regimen should be adjusted in time. Apalutamide, associated with endocrine disturbances and dermatologic reactions, necessitates regular monitoring of thyroid function, blood pressure, and skin evaluations, with dose interruption advised for severe cutaneous toxicity. Darolutamide is contraindicated in patients with hepatic impairment due to hepatotoxicity risks; baseline and bimonthly liver function tests are mandatory, and permanent discontinuation is recommended if hepatotoxicity develops. Enzalutamide, linked to neuropsychiatric AEs, warrants pre-treatment neurocognitive screening and periodic psychiatric assessments, with dose reduction or cessation for persistent symptoms. Prophylactic measures, such as photoprotection for dermatologic AEs and antiemetics for gastrointestinal toxicity, should be prioritized. Patient stratification by comorbidities and multidisciplinary collaboration are critical to optimizing safety.

Our research is based on the latest data from WHO-VigiAccess and is authoritative and timely. This study further analyzed the research trend of androgen antagonists through bibliometrics analysis, which has instructive significance. However, our study also has some limitations. First of all, there may be omissions or errors in adverse reactions reported in the WHO-VigiAccess. Meanwhile, most of the reports are from the Americas, which may be biased. Future studies should expand the sample size to improve the applicability of the conclusions. It is worth noting that there is no direct causal relationship between these adverse effects and androgen antagonists. Our research needs to be combined with the latest clinical trial results to guide more effective treatments. Recent studies have shown that patients treated with currently approved AR-targeted drugs develop resistance and relapse into castration-resistant prostate cancer (CRPC). In order to effectively inhibit reactivated AR signaling, new methods targeting AR should be actively explored. These new approaches include new small molecule inhibitors that target different domains of AR as well as drugs capable of degrading AR (Cole et al., 2024). Long-term studies are necessary to confirm potential adverse effects and develop personalized treatment.

## Conclusion

Androgen antagonists are crucial in prostate cancer treatment but have reported over 170,000 AEs globally. AEs mainly involve skin and subcutaneous tissue disorders and gastrointestinal disorders. Comparative analysis shows apalutamide may cause endocrine issues, such as rashes and hypertension. Darolutamide has notable hepatobiliary AEs, including hepatotoxicity. Enzalutamide is linked to nervous and psychiatric disorders. Most AEs are mild, but severe ones can be fatal. Future research should focus on pharmacogenomics to identify genetic factors for severe reactions like hepatotoxicity. AI-driven natural language processing can mine unstructured clinical data. Integrative omics approaches (proteomics, metabolomics, immunophenotyping) could reveal biomarkers for early detection of hypersensitivity or resistance, guiding personalized androgen antagonist selection and preemptive AE management.

## Data Availability

The original contributions presented in the study are included in the article/Supplementary Material, further inquiries can be directed to the corresponding authors.
